# Factors Affecting the Accuracy of Clinical Coding in the Casemix System in a Teaching Hospital: Cross-Sectional Study

**DOI:** 10.2196/93769

**Published:** 2026-09-17

**Authors:** Amirah Azzeri, Wan Mastura Wan Musaludin, Hafiz Jaafar, Nurnabihah MD Hafidz, Azimatun Noor Aizuddin

**Affiliations:** 1 Public Health Unit, Department of Primary Health Care, Faculty of Medicine and Health Ssciences, Universiti Sains Islam Malaysia, Persiaran Ilmu, Bandar Baru Nilai, Nilai, Negeri Sembilan, 71800, Malaysia; 2 Department of Public Health, Faculty of Medicine, Universiti Kebangsaan Malaysia, Jalan Yaacob Latif, Bandar Tun Razak, Cheras, Kuala Lumpur, Malaysia, 60 391702108

**Keywords:** clinical coding, casemix, diagnosis-related group, ICD-10, coding errors, coding accuracy

## Abstract

**Background:**

Accurate clinical coding is critical in the casemix system to ensure proper resource allocation, health care policy planning, and data reliability. Inaccurate coding can result in significant financial losses to the hospital. To date, the implications of inaccurate coding in casemix implementations in Malaysia have rarely been explored.

**Objective:**

This study aimed to evaluate the accuracy of clinical coding and identify factors associated with accurate coding practices in a major teaching hospital in Malaysia.

**Methods:**

A cross-sectional study was conducted using 445 inpatient discharge records from Hospital Canselor Tuanku Muhriz from January 2023 to December 2023. Stratified random sampling was applied across 4 departments. Coding accuracy was determined by comparing electronic medical record entries to a gold-standard set by trained coders and specialists. Descriptive and bivariate analyses were performed accordingly.

**Results:**

This study found that the overall clinical coding accuracy was 76.4% (340/445). Documentation completeness (*P*<.001) and coding turnaround time (*P*=.02) were significantly associated with coding accuracy. Other variables such as patients’ sex, patient age, department, and coder experience were not significantly associated. Clinical coding accuracy in this setting was suboptimal.

**Conclusions:**

Continuous training and regular audits are recommended to improve coding quality and ensure reliable casemix data for policy and funding decisions.

## Introduction

Clinical coding serves as the backbone of the casemix system, translating detailed patient diagnoses and procedures into standardized alphanumeric codes, such as those defined by the *International Classification of Diseases* and related classifications [1,2]. These codes are crucial for enabling workload analysis, hospital performance benchmarking, resource allocation, reimbursement processes, epidemiological research, and national health planning [2,3]. In Malaysia, the casemix system, specifically the Malaysian Diagnosis-Related Group (My-DRG), was first introduced at a major university hospital under the Ministry of Higher Education, aiming to improve efficiency and accountability in health care service delivery [4].

Despite the well-established role of casemix systems internationally, Malaysia’s nationwide implementation continues to face challenges, including shortages of trained coding professionals, inadequate infrastructure, and limited political and institutional commitment. One of the critical barriers to broader adoption is persistent concern over coding accuracy. Coding errors can lead to significant negative consequences such as financial discrepancies, misallocation of health care resources, flawed health policy decisions, and compromised data quality for research and planning [5,6]. Accurate clinical coding is especially vital in teaching hospitals, where patient caseloads are typically complex and involve multidisciplinary management.

The process of clinical coding is inherently intricate, involving the translation of complex diagnostic and procedural data into standardized codes while ensuring fidelity to clinical records. This process depends heavily on accurate and comprehensive documentation by health care providers, coders’ competence, and institutional systems supporting coding practices [7]. Studies have consistently demonstrated that coding errors arise from various sources, including incomplete or ambiguous medical documentation, coder experience and training gaps, and systemic factors such as departmental practices or weak organizational culture regarding data quality [7-9].

Several patient-related factors may influence coding accuracy. Older patients, for instance, often present with complex medical conditions and multiple comorbidities, making precise coding more difficult [10,11]. Similarly, longer hospital stays may indicate complex cases requiring multifaceted care, which can increase the potential for coding errors, although findings in the literature on this relationship remain mixed [12,13]. Departmental factors also play a role; surgical departments typically exhibit higher coding accuracy due to standardized procedural documentation, whereas medical and obstetric units face greater challenges due to diagnostic complexity and unplanned interventions [7,13].

Documentation quality is a critical determinant. Poorly written records, nonstandard abbreviations, or incomplete discharge summaries have all been linked to higher coding error rates [4,8]. Electronic medical records (EMRs), while intended to enhance documentation, can sometimes contribute to inaccuracies due to poor usability or reliance on default entries and copying-and-pasting practices [14,15]. Coder-related characteristics, including age, educational level, experience, and ongoing training, have also been shown to influence accuracy. Coders with higher qualifications and those who receive regular training tend to perform better [16-18]. In contrast, high workloads and time pressure are consistently associated with decreased accuracy [9,19].

Although international literature highlights these factors, research within the Malaysian context, particularly in teaching hospitals, is limited. Available studies suggest that coding accuracy levels in Malaysia vary widely, with error rates ranging from 25% to nearly 50% in different settings [6,7]. This variability underscores the need for localized investigations to identify modifiable factors that can enhance coding practices and data quality.

Therefore, this study aimed to determine the level of clinical coding accuracy and explore the patient, coder, documentation, and systemic factors associated with coding accuracy among discharged inpatients in a Malaysian teaching hospital. The findings will provide critical evidence to inform interventions aimed at improving coding practices, supporting effective casemix implementation, and strengthening the health system’s capacity for data-driven decision-making.

## Methods

### Overview

A cross-sectional study was conducted at Hospital Canselor Tuanku Muhriz (HCTM), a university hospital in Malaysia. It was formerly known as Universiti Kebangsaan Malaysia Medical Centre. It serves as a referral center for patients from Klang Valley and is also the first hospital in Malaysia that implemented a casemix system. HCTM requires coders to conduct active and ongoing diagnosis and procedure coding exercises.

This was a cross-sectional comparative study using secondary data obtained from inpatient discharge lists between January 2023 and December 2023. The aim was to assess the accuracy of clinical coding for the principal diagnoses. All patient information for the episode of admission was stored in the EMR system in the hospital database. In addition, the casemix unit at the International Centre for Casemix and Clinical Coding (ITCC) HCTM performed the diagnosis and procedure codings by extracting data from the EMR system. The database was accessed from June 1, 2024, to July 31, 2024. The authors had no access to information that could identify individual patients during or after data collection. Stratified random sampling was applied to obtain patients from 4 groups of departments: medicine, surgery, orthopedics, and obstetrics and gynecology. Subsequently, systematic random sampling was used via a computer system to select patient records from each department to ensure equivalent representativeness. The required sample size was calculated using the formula for finite populations, targeting a 95% confidence level and 5% margin of error. An estimated population of 31,103 discharges in 2023 yielded a required sample size of 384. A 20% buffer for incomplete data was added, yielding a final target of 445 records.

This study included secondary information from the discharge summaries in the EMR system, casemix-coded patient data, and coder demographic profiles. A comparison was made between the coders’ codes and the codes reassigned by a trained medical officer and casemix experts. The gold-standard codes were determined through a structured review process conducted by a trained medical officer with casemix coding experience and verified by certified casemix experts at HCTM. Each sampled record was independently reviewed against the discharge summary in the EMR, and the reassigned code was agreed upon through consensus between the medical officer and the casemix expert. In cases of disagreement, a third senior casemix expert served as the final arbiter to ensure a consistent and predefined adjudication process. To minimize assessment bias, the trained medical officer and casemix experts who reassigned the principal diagnosis codes were blinded to the original coder-assigned codes at the time of review. The original codes were only compared after the gold-standard codes had been independently assigned, ensuring that the reassignment process was not influenced by knowledge of the coders’ prior decisions. In this study, the dependent variable was coding accuracy, defined as whether the principal diagnosis was coded correctly by the casemix coder, identical to the codes assigned by the trained medical officer or casemix experts. The data were categorized into 2 groups: accurate or inaccurate. This study deliberately focused on the accuracy of the principal diagnosis code only rather than secondary diagnoses or procedure codes. This decision was based on the fact that the principal diagnosis is the primary driver of My-DRG casemix grouping and hospital reimbursement in the Malaysian casemix system, making it the most consequential code for financial and policy outcomes. It is important to note that this study evaluated the accuracy of the *International Classification of Diseases, Tenth Revision*, principal diagnosis coding specifically rather than the resulting My-DRG group assignment itself. My-DRG grouping accuracy depends on the joint accuracy of multiple coded elements (principal diagnosis, secondary diagnoses, procedures, age, and length of stay) and was beyond the scope of this study. Furthermore, restricting the analysis to the principal diagnosis allowed for a focused and interpretable measure of coding performance, consistent with several prior studies in similar health care settings [7].

Four components of independent variables were included: (1) patient-related factors such as age, sex, and length of stay; (2) clinical departments; (3) documentation factor; and (4) coder-related factors, namely, length of service, educational level, and time taken to assign a clinical code. The length of stay in hospital was defined as the number of days that the patient stayed in the hospital and was further divided into inliers and outliers. The discharge summary was reviewed for completeness of diagnosis. Documentation was considered complete if an actual primary diagnosis was recorded. Specifically, a discharge summary was considered complete if it contained an explicitly stated primary (principal) diagnosis written in full clinical terminology without reliance on abbreviations, symbols, or ambiguous shorthand. Summaries that contained only nonspecific descriptors (eg, “unwell,” “fever,” or “query diagnosis”), left the primary diagnosis field blank, or contained illegible entries were classified as incomplete. This operational definition was consistently applied by the reviewing medical officer across all sampled records.

For coder-related factors, the length of service was divided into less than 10 years and greater than or equal to 10 years. This threshold was used as it is consistent with the minimum reckonable service period for confirmed or pensionable status and optional retirement eligibility under Malaysia’s Pensions Act 1980 (Act 227), section 12 [20]. Educational level was categorized as having a degree or not, and the time taken for the coding officer to complete coding was calculated from the date of discharge until the date when the coding data were finalized. This period was divided into 2 categories: 3 days and below and 4 days and above. These cutoff points are consistent with the international discharged, not final coded benchmark used in health information management departments, where 3 days after discharge is the standard threshold for flagging a record as overdue for coding completion [21].

Descriptive analysis was conducted to present the background and study variables. Bivariate analyses were performed to understand the association between independent variables and the dependent variable. All associations were considered statistically significant if the *P* value was below .05. All data were recorded in Microsoft Excel and analyzed using SPSS (version 29; IBM Corp). Bivariate analysis using the Pearson chi-square test was performed to examine whether the coding accuracy status (accurate vs inaccurate) differed significantly across subgroups of each patient and coder characteristic. This approach was adopted to identify which specific variables were statistically associated with coding inaccuracy, thereby allowing for the prioritization of variables for targeted quality improvement interventions. In addition to the test of association, the coding accuracy rate for each variable category was calculated as the proportion of accurately coded records out of the total records in that category to provide a more interpretable measure of coding performance at the variable level. All 445 sampled records had complete data for discharge summary completeness status and coder-related variables. No records required exclusion due to missing data.

### Ethical Considerations

This study received ethics approval from the Medical Research and Ethics Committee of Universiti Kebangsaan Malaysia, with approval reference number JEP-2024-289. All patient data were anonymized to ensure participant confidentiality, and no individual participants could be identified during or after data collection. Informed consent was not required as the study used deidentified retrospective administrative data.

## Results

Of 445 inpatient records, this study found that 340 (76.4%) were coded correctly, whereas 105 (23.6%) were coded incorrectly. Of the 445 records included, 174 (39.1%) were of male patients, and 271 (60.9%) were of female patients. Regarding patient age range, the highest frequency was 30 to 39 years at 21.1% (n=94), followed by 60 to 69 years at 18.2% (n=81). The lowest frequency was in the ranges of 0 to 9 years and 10 to 19 years at 4.7% (n=21) each.

It was also found that 76.6% (341/445) of the inpatient records were categorized as inliers and 23.4% (104/445) were categorized as outliers. Among the 445 samples, the largest number corresponded to the medical department (n=123, 27.6%), followed by the obstetrics and gynecology department (n=110, 24.7%) and both the surgery and orthopedics departments (n=106, 23.8% each). Regarding the documentation of discharge summaries, 88.1% (392/445) of the records were complete, and 11.9% (53/445) were incomplete. Coders without a degree coded 28.1% (125/445) of the records, and those with a degree coded 71.9% (320/445). The number of records coded by coders with less or more than 10 years of service was almost equal. In terms of coding turnaround time, 72.1% (321/445) of the records were coded within 3 days or less, and 27.9% (124/445) were coded in 4 days or more. The descriptive analysis of patient sociodemographic and clinical characteristics and coder profiles is shown in Table 1.

**Table 1.** Descriptive analysis of patient sociodemographic and clinical characteristics and coder profiles (N=445).

Factors	Frequency, n (%)
Sex	
Male	174 (39.1)
Female	271 (60.9)
Age (y)	
0-9	21 (4.7)
10-19	21 (4.7)
20-29	50 (11.2)
30-39	94 (21.1)
40-49	50 (11.2)
50-59	38 (8.5)
60-69	81 (18.2)
70-79	59 (13.3)
≥80	31 (7)
Length of stay	
Inliers	341 (76.6)
Outliers	104 (23.4)
Department	
Medical	123 (27.6)
Surgical	106 (23.8)
Orthopedic	106 (23.8)
Obstetrics and gynecology	110 (24.7)
Coders’ educational level	
No degree	125 (28.1)
Degree and higher	320 (71.9)
Coders’ length of service (y)	
<10	227 (51)
>10	218 (49)
Time taken for coding (d)	
≤3	321 (72.1)
≥4	124 (27.9)

A total of 4 casemix coders were responsible for the 445 coded records included in this study. On average, each of them coded 100 to 110 cases. This uneven distribution reflects the actual workload allocation within the casemix unit at HCTM during the study period.

To determine which patient and coder characteristics were associated with coding accuracy, bivariate analyses were conducted alongside the calculation of accuracy rates per variable category. This dual approach allowed for identification of both the magnitude of coding accuracy for each subgroup and whether observed differences were statistically significant.

Table 2 shows the coding accuracy rate for each variable. Overall, 76.4% (340/445) of the records were coded accurately for the main diagnosis. At the variable level, coding accuracy varied across patient and coder characteristics. For sex, the accuracy rate was 79.9% (139/174) for male patients and 74.2% (201/271) for female patients, with no statistically significant association (*χ*^2^(1)=1.9; *P*=.17). Across age groups, accuracy ranged from 61.9% (13/21) in the group of 10 to 19 years to 84% (42/50) in the group of 40 to 49 years, although this variation was not statistically significant (*χ*^2^(8)=8.8; *P*=.36). For length of stay, accuracy was comparable between inliers (259/341, 76%) and outliers (81/104, 77.9%), with no significant difference (*χ*^2^(1)=0.2; *P*=.69).

**Table 2.** Coding accuracy by variable and association with accuracy status (N=445).

Factors	Accurate (n=340), n (%)	Inaccurate (n=105), n (%)	Chi-square (*df*)	*P* value
Sex			1.9 (1)	.17
Male	139 (40.9)	35 (33.3)		
Female	201 (59.1)	70 (66.7)		
Age (y)			8.8 (8)	.36
0‐9	15 (4.4)	6 (5.7)		
10‐19	13 (3.8)	8 (7.6)		
20‐29	39 (11.5)	11 (10.5)		
30‐39	63 (18.5)	31 (29.5)		
40‐49	42 (12.4)	8 (7.6)		
50‐59	28 (8.2)	10 (9.5)		
60‐69	66 (19.4)	15 (14.3)		
70‐79	47 (13.8)	12 (11.4)		
≥80	25 (7.4)	6 (5.7)		
Length of stay			0.2 (1)	.69
Inliers	259 (76.2)	82 (78.1)		
Outliers	81 (23.8)	23 (21.9)		
Department			3.6 (3)	.31
Medical	98 (28.8)	25 (23.8)		
Surgical	85 (25)	21 (20)		
Orthopedic	79 (23.2)	27 (25.7)		
Obstetrics and gynecology	78 (22.9)	32 (30.5)		
Discharge summary			42.4 (1)	<.001
Complete	319 (93.8)	73 (69.5)		
Not complete	21 (6.2)	32 (30.5)		
Coders’ educational level			0.1 (1)	.71
No degree	94 (27.6)	31 (29.5)		
Degree and above	246 (72.4)	74 (70.5)		
Coders’ length of service (y)			1.5 (1)	.23
<10	168 (49.4)	59 (56.2)		
>10	172 (50.6)	46 (43.8)		
Time taken for coding (d)			5.9 (1)	.02
≤3	255 (75)	66 (62.9)		
≥4	85 (25)	39 (37.1)		

Regarding clinical department, the highest coding accuracy was observed in the surgical (85/106, 80.2%) and medical (98/123, 79.7%) departments, whereas obstetrics and gynecology recorded the lowest accuracy rate at 70.9% (78/110), followed by orthopedics at 74.5% (79/106). However, these differences were not statistically significant (*χ*^2^(3)=3.6; *P*=.31).

The completeness of discharge summaries showed the most striking difference in accuracy rates. Records with complete discharge summaries had an accuracy rate of 81.4% (319/392) compared to only 40.4% (21/52) for those with incomplete summaries, a difference that was highly statistically significant (*χ*^2^(1)=42.4; *P*<.001). For coder-related factors, accuracy rates were similar between coders without a degree (94/125, 75.2%) and those with a degree or a higher educational level (246/320, 76.9%), with no significant association (*χ*^2^(1)=0.1; *P*=.71). Similarly, coders with less than 10 years of service had an accuracy rate of 74.0% (168/227) compared to 78.9% (172/218) for those with 10 or more years, which was not statistically significant (*χ*^2^(1)=1.5; *P*=.23). Finally, records coded within 3 days of discharge had a higher accuracy rate of 79.4% (255/321) compared to 68.5% (85/124) for those coded after 4 or more days, a difference that was statistically significant (*χ*^2^(1)=5.9; *P*=.02).

Overall, demographic and departmental factors did not significantly influence coding accuracy, whereas documentation completeness and timely coding emerged as key contributors. These findings highlight the importance of structured documentation practices and effective coder workflow management.

## Discussion

### Principal Findings

This study aimed to assess the accuracy of clinical coding for principal diagnoses at a major Malaysian teaching hospital. The findings revealed an overall coding accuracy rate of 76.4% (340/445). These results are consistent with those of prior literature but highlight room for improvement. For example, Olagundoye et al [22] reported a slightly higher accuracy rate of 78.7% at Lagos State University Teaching Hospital. Worldwide, clinical coding accuracy varies substantially, with a systematic review by Burns et al [23] identifying a median accuracy rate of 83.2%, ranging from as low as 50.5% to as high as 97.8%. Our findings fall within this range but remain below the optimal threshold for high-performing health systems.

Interestingly, our error rate was lower than the 49.8% reported by Zafirah et al [7] and comparable to the 25.2% observed by Saizan et al [6]. This difference may be attributed to methodological differences: while our study focused solely on the principal diagnosis, many others have evaluated multiple diagnoses and procedural codes, which are inherently more complex and prone to error. Such methodological clarity is a strength of our study, allowing for a focused and interpretable measure of accuracy that reflects core hospital performance in coding.

One of the most significant findings was the strong association between the completeness of discharge summaries and coding accuracy. In our sample, 88.1% (392/445) of the records were complete, substantially higher than the 7% completeness rate reported by Zafirah et al [7]. This likely stems from our focus on the principal diagnosis, whereas previous studies have assessed full diagnostic and procedural completeness. Nevertheless, our statistical analysis confirmed that complete documentation significantly improves coding accuracy (*P*<.001). These results align with those of Taiwo Adeleke et al [24], who reported that incomplete or poor-quality documentation was a leading contributor to coding inaccuracy in Nigerian hospitals. Moreover, Foster et al [5] emphasize that continuous auditing and feedback, combined with electronic discharge summary systems, can meaningfully enhance both documentation and coding standards. Active clinician engagement in the documentation process, as advocated by Nouraei et al [25], is also vital as the absence of clear diagnostic notes can critically impair the coder’s ability to assign appropriate codes.

Another notable finding was the statistically significant association between coding timeliness and accuracy. Records coded within 3 days of discharge were significantly more accurate than those coded after 4 or more days (75% vs 25%; *P*=.02). This supports the view that shorter coding turnaround times may reflect more efficient workflows or more straightforward cases. However, longer durations could also indicate increased care and attention to complex cases. Studies have found that coders who took additional time often achieved better accuracy, particularly for complex cases [26,27]. This raises the possibility that longer turnaround time may, in part, reflect greater case complexity rather than inefficient workflow alone, a distinction this study could not disentangle as case-level complexity (eg, number of comorbidities, diagnostic ambiguity, and length and diversity of documentation) was not independently measured. Coding complexity has been recognized elsewhere as a substantive, measurable construct that affects coder workload and throughput independent of coder efficiency [26,27], and future studies should consider incorporating a complexity measure (eg, number of secondary diagnoses, comorbidity index, or casemix index) to formally test whether case complexity confounds the turnaround time–accuracy relationship. Conversely, studies have also reported that prolonged delays might signal coder fatigue, systemic inefficiencies, or excessive workloads, factors that could compromise coding quality [28,29]. These findings suggest that turnaround time is a multifaceted indicator that could be used by hospital administrators to identify bottlenecks and improve workflow efficiency.

While the analysis of department-specific trends showed that obstetrics and gynecology had the highest rate of coding errors followed by orthopedics, internal medicine, and surgery, these differences were not statistically significant. Nevertheless, this trend aligns with findings by Zafirah et al [7] indicating that certain specialties may be more vulnerable to documentation and coding errors. This calls for department-specific interventions such as targeted coder training or closer clinician-coder collaboration, particularly in departments with complex case profiles.

Interestingly, coder-related factors such as educational attainment and years of experience did not significantly affect coding accuracy in this study. While this contrasts with literature suggesting that formal qualifications and experience enhance coder performance, our findings may be due to the small number of coders (n=4) and the relatively uniform training they receive at the ITCC. Future studies with larger and more diverse coder samples could provide more definitive insights into these relationships.

This study has several strengths. It is one of the few Malaysian investigations to focus specifically on principal diagnosis coding, providing a clear and interpretable metric of performance. It also combines administrative data with manually validated recoding, enhancing data reliability. Moreover, the statistically significant findings on discharge summary completeness and coding timeliness offer actionable insights for quality improvement.

However, some limitations must be acknowledged. This study was conducted at a single teaching hospital, limiting generalizability to other health care settings, including private or nonacademic hospitals. Teaching hospitals such as HCTM typically have access to specialist input; structured coder training programs; and more complex, tertiary-level case mixes than nonteaching public hospitals. Nevertheless, teaching hospitals may also differ from private hospitals in EMR infrastructure, documentation culture, and staffing ratios of coders to caseload. The coding accuracy patterns observed in this study, particularly the influence of documentation completeness and turnaround time, may therefore not directly generalize to district or state public hospitals or private health care settings, and multisite studies are recommended to confirm these findings across different hospital types.

Additionally, the small number of coders limited our ability to explore demographic factors robustly. With only 4 coders contributing to the 445 coded records, coder-level variables (such as educational level and years of service) were effectively compared across just 4 clusters rather than 445 independent units, substantially limiting statistical power to detect true coder-level effects. Nonsignificant associations for these variables should therefore be interpreted with caution rather than as confirmed null findings. This study also did not engage clinicians directly to understand root causes of documentation lapses, which could have enriched the analysis. Additionally, a formal interrater reliability assessment between the trained medical officer and casemix experts was not conducted, which is acknowledged as a limitation of this study. Future studies should incorporate a structured reliability assessment such as the Cohen κ to strengthen the validity of the gold-standard coding process.

It is acknowledged that the use of separate bivariate chi-square tests to examine associations between individual variables and coding accuracy status has limitations as this approach does not account for confounding between variables. In particular, potential confounders such as comorbidity burden, admission type (elective vs emergency), discharge disposition, and overall casemix severity were not captured in this dataset and could not be adjusted for. As this was a preliminary descriptive study designed to characterize bivariate associations rather than isolate independent predictors, these factors represent important variables for inclusion in future multivariable analyses. Future studies should consider using multivariable logistic regression, with coding accuracy status as the dependent variable and coder-related factors, including educational level, years of service, time taken for coding, and department, as independent variables. Such an approach would yield adjusted estimates and provide more robust evidence on the independent predictors of coding inaccuracy. In this analysis, chi-square tests were used to examine bivariate associations between coding accuracy and the variables of interest, rather than to identify independent predictors. The findings were intended to describe patterns of association and inform variables that may warrant further investigation in future multivariable analyses.

The analysis was limited to bivariate associations as an exploratory first step. A multivariable logistic regression model was not developed as this study aimed to describe preliminary associations rather than establish causal or predictive relationships. The small number of coders (n=4) further precluded robust multivariable modeling, and future studies with larger coder samples are encouraged to build on these findings. A multivariable logistic regression model was therefore not pursued at this stage as the primary aim was to describe the pattern of associations across variables rather than determine independent predictors. Future studies with larger and more diverse samples are recommended to perform logistic regression analysis to confirm these preliminary findings and identify independent predictors of coding inaccuracy.

Additionally, a sensitivity analysis restricting the sample to records with complete discharge summaries was not performed in this preliminary study. Given that documentation completeness was the strongest predictor of accuracy, future work should also examine whether other associations (eg, turnaround time and years of service) persist when analysis is restricted to records with complete documentation to rule out confounding by documentation completeness itself.

Despite these limitations, the findings have important implications for policymakers and hospital administrators. Improving the completeness and clarity of discharge summaries through training, digital templates, and clinical documentation improvement initiatives can directly enhance coding accuracy. In turn, more accurate coding enables better casemix classification, fairer hospital reimbursements, and more reliable health service planning. The significant relationship between coding timeliness and accuracy also suggests the need for balanced workload distribution and process optimization to reduce coding delays without compromising quality.

Hospitals should implement a structured, recurring coding audit program, for example, auditing 3.5% to 5% of monthly coded records on a weekly cycle, consistent with established health information management auditing practice, rather than relying on ad hoc or annual reviews [30]. Coder feedback should be delivered promptly after each audit cycle, with individualized scorecards highlighting recurring error patterns (eg, specific diagnosis categories or documentation gaps). A formal, structured clinician query process following established frameworks such as the American Health Information Management Association and Association of Clinical Documentation Integrity Specialists *Guidelines for Achieving a Compliant Query Practice* should be adopted to standardize how coders seek clarification from clinicians when discharge documentation is ambiguous or incomplete [30]. Standardized discharge summary templates with mandatory fields for principal diagnosis and key clinical indicators should also be piloted to reduce documentation-related coding errors, the single strongest predictor of inaccuracy identified in this study.

As health systems move toward performance-based financing and data-driven policy, coding accuracy becomes not merely a technical concern but also a strategic priority. In this context, this study’s findings offer a timely reminder that investments in documentation quality, coder support, and integrated clinical workflows are essential to realizing the full value of casemix systems in Malaysia and beyond.

### Conclusions

The results of this study showed that clinical coding accuracy was 76.4% (340/445), lower than the key performance index set by the Ministry of Health of Malaysia, which is 90%. The findings underscore the importance of complete documentation and timely coding in enhancing the accuracy of clinical coding in casemix systems. Hospitals should invest in ongoing coder training and implement systematic audits to ensure high-quality data, which is critical for planning, financing, and evaluating health care services.

## Abbreviations

EMR: electronic medical record
HCTM: Hospital Canselor Tuanku Muhriz
ITCC: International Centre for Casemix and Clinical Coding
My-DRG: Malaysian Diagnosis-Related Group
